# Telomere Length Reprogramming in Embryos and Stem Cells

**DOI:** 10.1155/2014/925121

**Published:** 2014-02-27

**Authors:** Keri Kalmbach, LeRoy G. Robinson, Fang Wang, Lin Liu, David Keefe

**Affiliations:** ^1^Department of Obstetrics and Gynecology, New York University Langone Medical Center, 180 Varick Street, No. 761, New York, NY 10014, USA; ^2^College of Life Sciences, Nankai University, Tianjin 300071, China

## Abstract

Telomeres protect and cap linear chromosome ends, yet these genomic buffers erode over an organism's lifespan. Short telomeres have been associated with many age-related conditions in humans, and genetic mutations resulting in short telomeres in humans manifest as syndromes of precocious aging. In women, telomere length limits a fertilized egg's capacity to develop into a healthy embryo. Thus, telomere length must be reset with each subsequent generation. Although telomerase is purportedly responsible for restoring telomere DNA, recent studies have elucidated the role of alternative telomeres lengthening mechanisms in the reprogramming of early embryos and stem cells, which we review here.

## 1. Introduction

Telomeres are tandem DNA repeats (TTAGGG_*n*_) which provide the substrate for specialized proteins to bind and loop chromosome ends. Telomeres prevent chromosome end-joining and ward off DNA damage repair machinery that would otherwise inappropriately repair the telomere as a double-stranded DNA break.

In humans, most tissues exhibit a marked decrease in telomere reserve over time [1, 2]. Cell division, oxidative damage, and genotoxic insults can directly or by way of DNA damage repair responses reduce the amount of DNA capping the chromosome end. Excessive telomere shortening induces cell senescence and eventually apoptosis [3]. Telomere DNA can be replenished by telomerase [3–5], a reverse-transcriptase-acting holoenzyme that adds a modest 50–100 bp per round of cell division. Expression of telomerase in humans is limited to highly proliferative tissues which harbor progenitor or stem cell compartments. In contrast to humans, many model organisms such as outbred mouse strains have constitutive telomerase expression coincident with longer telomeres. Recombination-mediated alternative lengthening of telomeres (ALT) pathway(s) have been proposed [6–8], which can augment telomere length by thousands of base-pairs within a few cell cycles [9].

Telomere biology is an emerging field that holds great promise for advancing clinical medicine, particularly for aging and age-related diseases. Short telomeres have been associated with the gamut of age-related diseases, including diabetes mellitus [10, 11], cardiovascular disease [12], liver disorders [13, 14], cancer [15–19], and death from all causes [20]. Moreover, long telomeres have been associated with exceptional longevity and increased lifespan [21]. Chemical or genetic depletion of telomere length recapitulates many of these pathologies in mouse models.

Direct evidence for the importance of telomere length in human disease derives from patients with mutations in *TERT* and *TERC*, the genes encoding the reverse transcriptase and RNA component of telomerase, respectively. Dyskeratosis Congenita, the prototypical telomere syndrome, results from mutations in the gene encoding many of the telomere maintenance genes, including *TERT*, *TERC*, *TINF2*, and DKC [16, 22–27]. This disorder manifests in tissues prone to high turnover, such as liver, fingernail beds, mucous membranes, and the hematopoietic system [16]. Symptoms arise when telomere lengths become critically short, which limits the ability of progenitor cells to maintain the differentiated cell populations in these tissues, leading to eventual organ system failure. Further, patients have a higher risk of developing cancer [28], particularly in those organs susceptible to telomere attrition. In families with inherited germline mutations in TERC and TERT, telomere length across generations is inversely related to the severity of disease manifestations. This genetic anticipation has also been documented in hereditary cancers [15].

Cellular reprogramming, resetting the aging process, requires purging of aged maternal proteins, degradation of maternal transcripts, resetting methylation cues, and rejuvenation of the genome and other cellular components. One paradigm to model this is somatic cell nuclear transfer (SCNT), where an adult cell is transplanted into an enucleated oocyte. Interestingly, though Dolly the Sheep, the first mammal cloned by SCNT, exhibited signs of precocious aging, presumably due to incompletely reset telomeres inherited from the donor somatic cell. Here, we will review *in vivo* and *in vitro* data on telomere length reprogramming, with particular emphasis on systems with the greatest promise for the future of personalized medicine.

## 2. Telomere Lengthening in Embryos

Mammalian oocytes arrest following the extrusion of the first polar body and prior to completion of meiosis II. Fertilization by a competent sperm leads to the extrusion of the second polar body, rendering the oocyte haploid to complement the incoming paternal genome. What is known about telomere dynamics during this period is largely limited to observational studies, both in mouse and human.

Telomere length in the oocyte is markedly shorter than somatic cells [9, 29–31], which is particularly intriguing given that the majority of an oocyte's lifespan is spent in meiosis, when replication-linked telomere attrition is thought not to have an influence. High reactive oxygen species, generalized aging, and lack of telomerase activity conspire to reduce telomere length in oocytes [30, 32, 33]. In contrast, sperm are of the few cell types documented to elongate telomeres over the human lifespan [34–37], presumably due to the effects of telomerase activity in spermatogonia throughout the life of the male.

Following fertilization and activation of the egg, embryonic cells undergo dramatic telomere lengthening [9, 31, 38, 39]. In mouse, telomere length increases by thousands of base-pairs within the first one or two cell cycles [9], nearly doubling from the baseline oocyte length to the two-cell stage. Notably, telomerase activity remains undetectable in these cells. Instead, embryos exhibit high rates of telomere sister chromatid exchange (T-SCE), telomere-specific localization of recombination proteins, and a favorable chromatin state. This effect remains robust in telomerase knock-out mice, suggesting an ALT-dependent mechanism at play in preimplantation mammalian development. Moreover, the lengthening takes place in parthenogenetically activated eggs, which lack sperm input during activation, suggesting that the capacity for telomere length reprogramming resides in the oocyte.

**Figure 1:**
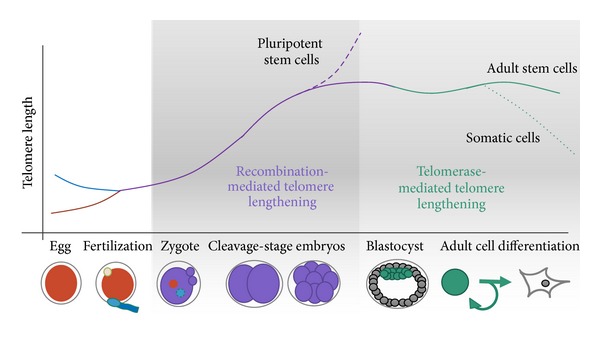
Schematic of telomere length reprogramming in mammalian embryonic development. The greatest telomere lengthening takes place during the earliest stages of preimplantation development, the cleavage-stage embryo, which may coincide with zygote genome activation. Recombination-mediated telomere lengthening (ALT) is also purportedly responsible for reprogramming in pluripotent stem cells, including ESCs, ntESCs, and iPSCs. Later, in development and adult life, telomerase becomes the dominant telomere maintenance mechanism for the inner cell mass and in tissue-specific telomere replenishment in stem cell niches.

Whereas telomerase-independent, recombination-based telomere elongation takes place during early preimplantation development, telomerase activity increases dramatically at the blastocyst stage of development (see Figure 1). The greatest degree of telomere lengthening occurs in the inner cell mass, the pluripotent cells giving rise to the embryo proper [40], in contrast to the trophectoderm, which gives rise to tissues that will become placenta and other extra embryonic tissues. Interestingly, telomere length in the inner cell mass is substantially shorter than that of embryonic stem cells, which lengthen after several passages in culture. Studies of telomere length dynamics during early embryo development and blastocyst development and during formation of ESCs should be conducted on human tissue.

Owing to the limitations in the United States for using human embryos in research, these studies have not been directly repeated in humans. However, one group based in the United Kingdom attempted to resolve telomere length at the level of the individual chromosome by utilizing semiquantitative fluorescent in situ hybridization (QFISH) with individual gametes and pronuclear embryos [41, 42]. This group found that telomere length is shorter in sperm than oocytes, including in immature oocytes and mature oocytes and at the pronuclear stage. The results of these experiments are open to interpretation for several reasons. Until fertilization, immature and mature oocytes contain twice the amount of DNA as sperm. Moreover, QFISH usually requires metaphase spreads to measure telomere length, so the use of individual interphase spreads needs to be validated. Sperm and oocyte telomere length discrepancies found by this method could also be accounted for by differing chromatin states that allow the probe to have more ready access to the oocyte pronucleus over the highly condensed male pronucleus. Methods which account for DNA copy number, such as single cell qPCR [31], could correct for these shortcomings.

Assisted reproductive technologies afford the opportunity to directly access human gametes and embryos, allowing for such studies of telomere length dynamics during human preimplantation development. Such studies have revealed shorter telomeres in oocytes of women who do not conceive following IVF compared to those who do [29] and in oocytes from cycles producing fragmented embryos [43]. Moreover, shorter telomere length was found in aneuploid blastomeres and polar bodies than in euploid cells from the same IVF patient and cycle [39], which is consistent with similar findings in mouse models with short telomeres [44]. Indeed, a wide range of complications of advanced reproductive age have been associated with shorter telomere length, including Down syndrome [45] and recurrent miscarriage [46] (also reviewed in [30]).

Although the studies provide a unique insight into events during telomere reprogramming, these studies by necessity remain observational. Interestingly, a variety of studies have revealed basic reprogramming capacity in offspring from somatic cell nuclear transfer (SCNT), which creates a cloned embryo, such as Dolly the Sheep. Since the telomeres in somatic cells in most mammalian species exhibit age-related decline, use of SCNT-cloned animals provides an interesting sample of reprogramming across species. Newborn clones of pig and cattle exhibit normal telomere lengthening during embryonic reprogramming, although the rate of production of viable, full-term offspring by way of SCNT is very low [47–51]. Minor discrepancies in the degree of telomere lengthening amongst these studies might be explained by the small sample sizes and the differential time points of tissue selection for telomere length analysis. Overall, these studies are consistent with observational studies in human and experimental studies in mouse that demonstrated a minimum telomere length is likely required for the development of a competent embryo; to determine this experimentally, it would be intriguing to increase telomere length and examine efficiency of SCNT and production of cloned embryos.

## 3. Telomere Length Reprogramming in Stem Cells

Stem cells are defined not only by their differentiation potential but also by their capacity for unlimited self-replication. The need for prolonged self-replication requires adequate telomere length and telomere maintenance, which can limit the powerful new methods available for generating induced pluripotent cells. IPSCs lacking sufficient telomere length fail to achieve germline transmission or tetraploid complementation, the most stringent tests of pluripotency, and cannot be maintained in culture over long periods. This might have contributed, in part, to the variable quality of iPSCs during early efforts by the Yamanaka group and the initial failure of these cells to contribute to chimeras and may ultimately limit the future application of iPSCs in regenerative medicine [52, 53]. To correct this, present efforts in the field of iPSCs have strived to improve the quality of iPSC generated by focusing on telomere dynamics during the process of reprogramming.

Late passage, telomerase-deficient (*Terc* ^−*/*−^) murine embryonic stem cells (ESC) possessing critically short telomeres show reduced cell growth, reduced ability to form teratomas, and failure to transmit through the germline to form chimeras [54]. Since expression of the common pluripotency markers (Oct4, Nanog, Sox2, Lin28, etc.) does not differ between telomerase-deficient and wild type ESCs, telomere length may provide a more sensitive measure of pluripotency. Further work generated telomerase haploinsufficient and deficient (*Terc* ^−*/*+^ and *Terc* ^−*/*−^) iPSCs, revealing telomerase dispensability during the reprogramming process while also demonstrating the requirement for telomerase to maintain telomeres and pluripotency in iPSC during culture [55]. Identifying lack of true pluripotency in iPSC with short telomeres recapitulated earlier suggestions linking telomere length with detection of pluripotent status. This study also revealed an increased rate of telomere sister chromatid exchange (T-SCE) in the absence of full telomerase function, demonstrating a telomerase-independent mechanism (ALT) at work during reprogramming. In particular, this study implicated limited telomerase function (possibly arising from poor activation by pluripotency factors) and reactivation of exogenous pluripotency factors in response to loss of full telomerase function, in addition to ALT in contributing to telomere length variability in iPSC reprogramming.

Recombination events such as T-SCE require reductions in DNA methylation and epigenetic remodeling and are frequently associated with genomic stability [56]. Understanding the epigenetic mechanisms regulating telomere length is critical for improving the quality of iPSC. Critically short telomeres in a telomerase-deficient (*Terc* ^−*/*−^) ESC model exhibited reduced global hypomethylation and altered levels of H3k27_me3_, which led to increased *Nanog* and *Gata 6 *expression, inducing reversible differentiation [57]. Rescue of stable differentiation was achieved by restoration of Dnmt3b or inhibition of *Nanog* resulting inreversed global DNA hypomethylation and normal repression of *Nanog* by de novo methylation. This work led to a model in which adequate telomere length directly impacts the ability of cells to maintain stable differentiation capacity.

*Zscan4* is emerging as a potentially important factor in generating high-quality iPSCs. First identified for its essential role in development from 2-cell to 4-cell embryos [58], *ZScan4* was later shown to play a role in ESC regulation. Importantly, a Zscan4-positive state has been observed in ESC populations, where it colocalizes to telomeres and accompanies telomere elongation [59]. Moreover, *ZScan4* knockdown accompanied reduced proliferation, decreased T-SCE, decreased telomere length, and abnormal karyotype. Over subsequent passages, the majority of Zscan4-deficient cells became cell cycle arrested. Taken together, these findings point to an essential role for *Zscan4* in regulation of telomere length in order to perhaps protect cells from genomic instability. Interestingly, the addition of *ZScan4* to Yamanaka factor reprogramming produced tetraploid-complementation competent cells with longer telomeres than traditional iPSC, enabling the formation of chimeras from over 50% of cell lines generated. More recent work has elucidated the regulatory relationship between *Tbx3 *and *Zscan4*, namely, that *Tbx3* can activate *Zscan4 *by inhibiting DNA methylation at subtelomeric regions, leading to depression of genes in this region (e.g., *Zscan4*) [60]. The addition of *Zscan4 *or *Tbx3* to the reprogramming protocol produces iPSC able to pass the most stringent tests of pluripotency and pushes closer toward the development of a protocol for iPSC suitable for clinical application [61].

In humans, the nature of telomere reprogramming has recapitulated many findings in other model organisms. Generation of human iPSC (hiPSC) from patient cells with the telomere disorder Dyskeratosis Congenita demonstrated the ability to elongate telomeres after reprogramming [62]. While in early passages these dyskerin-mutated (*DKC1*) hiPSC lines showed gradual telomere reduction, prolonged culture enabled telomere resetting to match the length of parent fibroblasts, indicating that the *TERC* deficiency within these cells had been overcome by the reprograming process. Other work with *DKC1* hiPSC revealed that extended culture led to telomere shortening and spontaneous differentiation [63]. Additionally, hiPSC from patients with mutations in telomerase itself (*TCAB1*) that cause mislocalization of the enzyme fails to lengthen telomeres despite having normal telomerase activity, indicating that proper localization is as critical a factor as activity for telomere maintenance. Combined, these data reinforce that many factors affecting telomere length and telomere maintenance ultimately impact pluripotency and self-renewal of human and animal cells.

SCNT would provide the gold standard for production of personalized stem cells for humans, as the resulting progeny are veritable clones of the donor genome. Although methods for deriving SCNT stem cells were conceived over a decade ago, this was only successfully completed in humans very recently due to excessive technical difficulty and ethical limitations [64]. Recently, a direct comparative study between SCNT derived cells (ntESC) and iPSC from telomerase-deficient (*Terc* ^−/−^) mice has explicitly determined that SCNT significantly increases telomere lengthening during reprogramming and improves differentiation capacity and proliferative ability relative to Yamanaka reprogramming. The data suggest that SCNT produces superior pluripotent cells and that the process can rescue differentiation ability in cells with critically short telomeres. Still, the requirement for high-quality human oocytes for SCNT will likely drive more in-depth studies into the factors within the oocyte necessary to produce superior stem cells [65]. These factors, once isolated, will likely have an explosive impact on Yamanaka reprogramming both in terms of the quality of the cells and the efficiency of the process.

## 4. Discussion

Advances in modern medicine have led to remarkable prolongation of human life span. Concomitantly, an increase in delayed child rearing until later in life has led to reduced fecundity. As a result, the burden of age-related diseases and infertility makes elucidation of the pathobiology of aging an urgent priority.

IPSCs bypass the ethnical and immune-compatibility issues that limit application of embryonic stem cells for therapeutic purposes. Not only do these iPSCs promise to provide advanced models and treatments of complex human diseases, but the efficiency of reprogramming, especially pertaining to telomeres, must be fully addressed before clinical applications can be considered.

In human reproduction, a minimum telomere length is likely required for production of a competent embryo, as in the development of pluripotent cell lines. Lessons from reproduction, such as the discovery of the ZScan4 factor, may help increase the efficiency of reprogramming methods and the clinical practicality of the resulting cells. In particular, members of the shelterin complex have been shown to be critical for reprogramming efficiency [66, 67]. For instance, while *TRF1 *expression level cannot be correlated with telomere length, the presence of *TRF1* is essential for induction and maintenance of pluripotency in iPSC, in addition to significant correlations between the level *TRF1 *and the level of potency in the resulting cells. Moreover, epigenetic states of the subtelomere seem to positively regulate telomere lengthening through recombination [68–70] and may someday improve reprogramming efficiency. Expanding our knowledge of the basic biology telomeres may hold the key to correcting age-related infertility and other age-related diseases.
